# Evaluation of Articular Eminence Inclination in Normo-Divergent Subjects with Different Skeletal Classes through CBCT

**DOI:** 10.3390/ijerph18115992

**Published:** 2021-06-03

**Authors:** Francesco Moscagiuri, Francesco Caroccia, Chiara Lopes, Beatrice Di Carlo, Erica Di Maria, Felice Festa, Michele D’Attilio

**Affiliations:** Department of Innovative Technologies in Medicine & Dentistry, University of Chieti-Pescara, 66100 Chieti, Italy; francesco.moscagiuri@unich.it (F.M.); fcaroccia20@gmail.com (F.C.); chiaralopes17@gmail.com (C.L.); bdc.dicarlo@gmail.com (B.D.C.); erica.dim95@gmail.com (E.D.M.); felice.festa@unich.it (F.F.)

**Keywords:** temporomandibular joint, computed tomography, CBCT, articular eminence inclination

## Abstract

(1) We aimed to compare articular eminence inclination (AEI) in normo-divergent subjects (SN^GoGn = 32° ± 5°), with different anteroposterior sagittal skeletal classes through a cone beam computed generated tomography (CBCT). (2) In this cross-sectional study, CBCT records were retrospectively analysed. From the original sample of 52 CBCT records, 33 records of normo-divergent adult subjects were selected (11 Class I, 13 Class II and 9 Class III). On mid-sagittal section of the Temporomandibular Joint (TMJ) on both sides, AEI was calculated by graphic method. (3) The Kruskal–Wallis test was used to evaluate any difference between AEI on both left and right sides in the three groups. No statistically significant difference was observed on either the right side (*p* = 0.174) or the left side (*p* = 0.624). (4) Articular eminence inclination seems to be not related with skeletal class. Given the lack of significance in the observed differences between AEI and skeletal classes, we believe that future studies should focus on assessing possible relationships between AEI and different vertical skeletal patterns.

## 1. Introduction

### 1.1. Background

The temporomandibular joint (TMJ) allows the jaw to articulate with the rest of the skull. The TMJ is formed, inferiorly, by the mandibular condyle and superiorly, by the temporal bone [1]. Functional forces influence its morphology [2] and the different skeletal classes can influence its components such as the mandibular condyle and glenoid fossa [3]. The articular eminence (AE), situated on the temporal bone, is an important part of the TMJ and represents the anterior limit of the glenoid fossa. The AE should not be confused with the articular tubercle, another structure on the lateral side of the AE, where the temporomandibular ligament takes origin [4]. AE morphology is described as convex with different inclinations in different people [5]. The articular eminence inclination (AEI) is an angle formed by the articular eminence and the Frankfort Horizontal (FH) plane or any other horizontal plane [4]. To delimit this angle, the plane that surrounds AE could be taken from the best-fit line on the slope of the eminence, or with a line connecting the roof of the fossa with the highest point of eminence [4]. Both planes define the AEI, but while the former focuses on the posterior surface of the eminence, the latter better describes the location of the eminence crest relative to the fossa roof. The normal angle size ranges from 30° to 60° [4]. Inclinations lower than 30°, are defined as flat, while those with values greater than 60° are defined as steep. However, this classification is not universally accepted. Several authors classify articular eminence inclinations based on subjective criteria and divide them into flat, moderate and protuberant [4]. The movement of the condyle inside the fossa is influenced by the slope of the articular eminence. The condylar path is more vertical if the AE slope is steep, while it is less vertical when it is flat [6]. AE features, including shape, influence mandibular movement, which is also conditioned by dental absence [4,7], age [4,8,9], skeletal malocclusion [5,10], sex [8,11], and masticatory loads [12]. AE morphological variations may lead to TMJ mechanical alterations and may function as predisposing factors to internal dysfunctions [13,14], such as anterior disk displacements (ADD) [1]. However, there is no difference in the AE angle between joints with ADD with reduction and those with ADD without reduction [15]. Moreover, the vast majority of studies failed in demonstrating a clear relationship between the AE angle and the severity of temporomandibular disorders using CBCT [1]. AEI could also be influenced by degenerative bone diseases (DBD) and by the shape of the mandibular condyle. Several radiographic exams, including magnetic resonance imaging (MRI), computerized tomography (CT) scan and cone beam computed generated tomography (CBCT) could be used to visualize the TMJ and assess AEI [16]. CBCT scans seem to give an extreme reliability, precision and reproducibility in the identification of landmarks, including bilateral ones, compared to digital cephalograms [17]. Moreover, CBCT is likely to be the best choice for the visualization of TMJ osseous morphology [1,18] as it offers high-resolution three-dimensional images without magnification or distortion [18]. A number of studies attempted to assess whether vertical and sagittal malocclusions have an influence on TMJ structural features. However, outcomes of these studies remain discordant. The vertical skeletal pattern has a greater influence on the TMJ than the sagittal skeletal type [19,20] and this is important in establishing proper treatment for the temporomandibular disorder. Some studies showed that gender and sagittal as well as vertical skeletal patterns affect condylar height and volume, with higher values among men, class III, and hypodivergent patients [20]. Some authors observed that condylar height and width were increased on both sides from class II, I and III [9]. Concerning the vertical pattern, various studies investigated how it could affect TMJ structures. Some studies showed that AEI was greater in males with brachycephaly compared to other facial types [12], while others reported that hypodivergent patients have a bigger condylar width than normodivergent and hyperdivergent subjects [9]. Hyperdivergent subjects also appear to have smaller condylar anteroposterior diameters compared to normodivergent and hypodivergent patients [18]. Furthermore, these subjects show a more anterior position of the mandibular condyles compared to normodivergent and low angle ones [19]. Conversely, another study reported that a vertical malocclusion does not affect mandibular condylar head size, its perimeter, area, height, and surface shape [10]. Asymmetry between right and left TMJs among the same subject has also been reported [21]. Finally, Arieta-Miranda et al. reported differences in both condylar position and articular eminence angle in subjects with different sagittal class. However, they did not consider the same vertical pattern for all the subjects [8]. 

Studies investigating exclusively how sagittal skeletal patterns affect AEI often have reported conflicting outcomes. Sagittal skeletal malocclusion and age could modify condylar and fossa shape, position of TMJ structures and AEI [2], with lower values in class III individuals [22]. Paknahad et al. reported a correlation between position of the condyle and skeletal class in a normodivergent group of subjects without investigating the AEI [23].

### 1.2. Objectives

Following a group formation similar to that reported by Paknahad et al. [23], our study aims to evaluate AEI in a group of normodivergent (SN^GoGn = 32° ± 5°) subjects subdivided according to the sagittal skeletal pattern with the scope of identifying a possible and specific correlation between sagittal but not vertical pattern and AEI. 

## 2. Materials and Methods

This is an observational retrospective study conducted on CBCT records. The sample size includes 52 adult subjects, 24 males and 28 females, aged between 21 and 35 years (average age 25 ± 1.4 years), who visited the Department of Innovative Technologies in Medicine & Dentistry of the University of Chieti-Pescara, Chieti, Italy from January 2014 until December 2019. These patients were submitted to CBCT imaging during their orthodontic and gnathological treatment. Data recorded in these instances have been subsequently used to conduct the study. 

The study was conducted in observance of the Helsinki Declaration (revised version of Tokyo in 2004) and Good Clinical Practice Guidelines. The STROBE statement has been followed in the reporting of this study [24].

To meet the inclusion criteria the medical history of every subject was collected from the Departmental archive. CBCT records without an exhaustive medical history were excluded. Only subjects who met the following inclusion criteria were recruited and their CBCT records collected: no history of craniofacial trauma or fracture, no indications or symptoms of temporomandibular disorders, full permanent dentition (except third molars that were extracted or with agenesis), no previous orthodontic treatment or orthognathic surgery, no congenital craniofacial syndrome diagnosis, no facial asymmetry, no condylar hyperplasia, no systemic disease affecting TMJ (i.e., arthritis).

CBCT (Vatechlpax 3D PCH-6500, Fort Lee, NJ, USA) [25] was performed respecting these parameters: 5.0–9.0 mA (it depends on the age of the patient), 80–110 kV (it depends on the age and size of the patient), 24 s of scan time, large sized FOV of 24 cm × 19 cm. During the CBCT exam the patient’s head was oriented with the Frankfurt plane (Po-Or) parallel to the floor and perpendicular to the sagittal midline previously located on the axial plane, connecting opisthion (Op) and crista galli (Cg); no support for the chin was used. The subjects were clearly informed about the radiographic procedure; they were asked to avoid any type of movements and keep centric occlusion. Before leaving the X-ray room, the operator confirmed the centric occlusion and invited the subject to occlude with the lip in light contact. After X-ray scanning, DICOM imagine files were processed by Ez3D Plus Software (Vatech, Global Fort Lee, NJ, USA) [25]. For each subject, lateral cephalograms were extracted. 

Using the cephalometric software OrisCeph3, an expert operator traced the cephalometric measurement of each subject (Table 1). To test the examiner’s internal reproducibility, after performing 30 cephalometric measurements, we randomly selected 5 to be re-examined to guarantee internal agreement calculating Cohen’s kappa coefficient (*κ*). Two independent expert orthodontists analysed the data extracted from cephalometric measurements. Facial divergence of the subjects was evaluated with SN^GoGn angle: the confidence interval considered was 32° ± 5°, and for values lower than 27° and higher than 37°, subjects were judged as hypodivergent or hyperdivergent, respectively. Thirty-three subjects were judged normodivergent and continued the study.

Therefore, two expert orthodontists divided the 33 normodivergent subjects according to the sagittal skeletal class. Subjects were so divided into Class I, Class II or Class III using the integrated evaluation of the following cephalometric measurements: ANB angle, reconstructed Wits index, distance of points A and Pogonion from McNamara line (a line perpendicular to the Frankfurt plane crossing skeletal Nasion point) and incisors inclination (Table 1). Any difference in the skeletal class evaluation between the two orthodontists was discussed to achieve an agreement.

**Figure 1:**
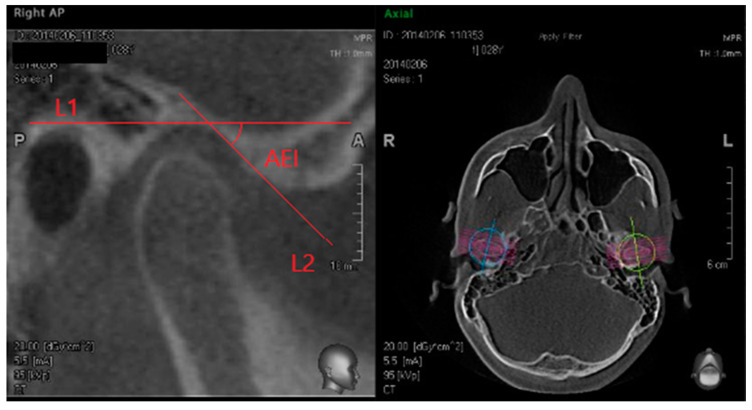
Graphic method to measure Articular Eminence Inclination. The mid-sagittal section of the condyle was separately extracted on the right and left. On the axial view, the section of the condylar process that had the widest mediolateral diameter was chosen as the reference view for reconstruction of the sagittal slices. In this section, a line orthogonal to the mediolateral diameter of the condyle and parallel to the long axis of the condylar process was drawn, so sagittal images were reconstructed as 0.5 mm slice interval/thickness and the most central one was taken. Legend: L1: line 1, a horizontal line, parallel to the Frankfurt plane (FH) passing through the uppermost point of the glenoid fossa; L2: line 2, constructed along the posterior slope of articular eminence, connecting the lowermost and most posterior point of the articular eminence and the uppermost and most anterior point of the glenoid fossa on the temporal bone; AEI: Articular Eminence Inclination, the angle measured between the two lines.

From the CBCT records the mid-sagittal section of the TMJ on both sides was also extracted. On the axial view, the section of the condylar process that had the widest mediolateral diameter was chosen as the reference view for reconstruction of the sagittal slices. This axial section was separately chosen on the right and left side. In this section, a line orthogonal to the mediolateral diameter of the condyle and parallel to the long axis of the condylar process was drawn, so sagittal images were reconstructed as 0.5 mm slice interval/thickness. The measurements were established on the central sagittal section of the condyle. This protocol was used to orientate and position the tomogram cut to be perpendicular to the long axis of each condyle and at the centre of the condyle (Supplementary Materials Figure S1). On this mid-sagittal section, two lines were traced (Figure 1):

- L1: a first horizontal line, parallel to the Frankfurt plane (FH) passing through the uppermost point of the glenoid fossa.
- L2: a second line, constructed along the posterior slope of articular eminence, connecting the lowermost and most posterior point of the articular eminence and the uppermost and most anterior point of the glenoid fossa on the temporal bone.

The angle included between the two lines describes the articular eminence inclination (AEI) on the sagittal plane. All AEI angles were measured twice with a 2-week washout interval after the first measurement to assess the significance of any error during measurements with Intraclass Correlation Coefficient (ICC).

### Statistical Analysis

Statistical calculation was made by using SPSS software (SPSS Inc. software, Chicago, IL, USA). The Cohen’s kappa coefficient (*κ*) was used to confirm intra-observer reliability for the cephalometric measurements and Intraclass Correlation Coefficient (ICC) was used for the AEI measurements. The Shapiro–Wilk normality test was applied to check whether data were normally distributed. Since data were not normally distributed, non-parametric tests were applied. The Friedman test was used to compare the mean difference between right AEI and left AEI in each skeletal class. The Kruskal–Wallis test was used to evaluate any difference between AEI in the three groups on the left and right side. The present study judged a *p*-value less than 0.05 as significant.

## 3. Results

According to the integrated cephalometric analysis, the 33 normodivergent subjects were divided into three groups with different skeletal classes (Table 2): 11 subjects (5 male and 6 female) were evaluated as Class I, 13 (6 male and 7 female) as Class II and 9 (4 male and 5 female) as Class III. The Cohen’s kappa coefficient (*κ*) showed high agreement of the correlation coefficients for all the cephalometric parameters with an average value of 0.95 (0.88–0.98). The values for ICCs for AEI values varied from 0.720 to 0.993, indicating substantial intra-rater agreement.

Measurements of articular eminence inclination angle referring to both right and left sides for the three groups are reported in Table 2. 

The Friedman test was used to compare the mean difference between the right AEI and left AEI in each skeletal class. For skeletal class I, no statistically significant difference was found between AEI on the left and right side. Conversely, statistically significant differences between the two sides were found in both class II (*p* = 0.004) and class III (*p* = 0.020). Since statistically significant differences were found between right and left temporomandibular joint measurements, the data from the two joints for all the three groups were not pooled together and the analysis was conducted independently on the left and the right side.

The Kruskall–Wallis test (Table 3) was used to evaluate any difference between AEI in the three groups on the left and right side. No statistically significant difference was observed on the right side (*p* = 0.174) and on the left side (*p* = 0.624).

## 4. Discussion

The present study aimed to evaluate the relation between sagittal skeletal malocclusion and AEI in subjects presenting with a normodivergent vertical pattern on CBCT records. The results showed that there is no statistically significant difference. 

Articular eminence inclination indicates condylar guidance and can be measured either by clinical (i.e., occlusal wax registration, intraoral equipment for registration, etc.) or radiographic methods. According to the literature, radiographic methods are better reproducible and can be standardized more easily than clinical methods [26]. Radiographic methods allow the selection of specific reference points on radiograms resulting in reproducible and standardizable data. However, the disadvantage of using radiographic methods relies on the potentially harmful exposure of the patient to x-ray radiation. In this study, AEI was calculated by radiographic methods on mid-sagittal section of the TMJ extracted from a CBCT. 

In these studies, AEI values range between 22° and 64°. These results are in line with those previously described in the literature [4,27,28]. Indeed, Christiansen et al. [29] and Lobo et al. [22] reported an AEI normal value for adults ranging between 30° and 60° and 39.25° to 55.42°, respectively. Conversely, these results appear to be significantly different from those reported in other studies [5,11] and from the range of 42° to 58° described by Arieta-Miranda [8]. Additionally, the AEI mean value observed in each skeletal class was different from the one reported by Arieta-Miranda [8]. The latter observed that articular angle was higher in the Class I group (58°), decreased to 51° in Class II, and was even lower in the Class III group (42°). Our investigation, however, observed no statistically significant difference within the three skeletal classes with mean values of 44.45° for Class I, 44.50° for Class II and 40.75° for Class III. The mean age of our sample and the radiographic method applied to estimate AEI, are superimposable to those in the investigation by Arieta-Miranda et al. [8]. However, in the study by Arieta-Miranda, the sample was not classified according to the vertical skeletal pattern and this could explain the observed difference. Moreover, the same sample showed an increased vertical facial pattern in groups of the Class II and III while our study examined only normodivergent subjects within the three groups.

There are only a few studies correlating the skeletal sagittal class and the AEI and none of them isolated the variable vertical pattern. Lobo et al. [22] found no significant difference between Classes I and II, confirming the results of our study. Concerning Class III, they observed significant lower values [22], while this study observed a mean value in the Class III pattern (40.75°) lower than that in Class I (44.45°) and II (44.50°). However even in our case, these differences did not reach statistical significance. 

Ikai et al. [30] measured dry skulls and reported a negative correlation between the angle of the eminence and the ANB angle. Both Katsavrias et al. [2], investigating digitized tomograms, and Akahane et al. [31], investigating dry skulls, found a smaller eminence to FH angle in the Class III group. Singh et al. [6] traced AEI with one clinical method and two radiographic methods on lateral cephalograms. One of the radiographic methods, the tangent method [6] is comparable to our method and showed that the Class III group had a significantly lower angle (37.60°) compared to Class I (41.90°) and Class II (43.90°). These results seem to be opposite to those of our study. Possible reasons for these apparent discrepancies could rely on the fact that their measurements were taken on dry skulls [30,31] or through tomograms [2] and, in addition, none of these studies considered the vertical pattern.

Krisjane et al. [32] investigated TMJ morphology in Class II and Class III subjects on 3D CT records measuring the AEI with an angle between the plane of the posterior wall of the articular tubercle and the plane obtained from the most inferior point of the articular tubercle to the most inferior point of the auditory meatus. Although a different measurement method was used and the vertical pattern was not considered [32], they observed no statistically significant difference between the sagittal patterns, thus confirming our results. 

### Limitations

This study presents some limitations that have to be pointed out. The main limitation is that the central cut of the condyle would be the most representative section for analyzing condylar position and articular eminence shape but it is difficult to identify it. Indeed, the anatomy of the structures varies in different sections of the joint complicating its identification even in a three-dimensional radiograph. Since no power analysis was done and since the sample size might be slightly small, the present study can be considered as a pilot study. Finally, the graphic model for AEI calculation selected here should be validated on a different sample. Future studies assessing the relation between the AEI and the sagittal or the vertical skeletal pattern may benefit from using samples of a larger size.

## 5. Conclusions

Articular eminence inclination seems to not be related to skeletal class. Given the lack of significance in the observed differences between AEI and skeletal classes, we believe that, in the future, it would be interesting to assess whether possible relationships exist between AEI and different vertical skeletal patterns.

## Figures and Tables

**Table 1:** Mean value and confidence interval of the cephalometric parameters.

Cephalometric Parameter	Mean Value		
SNA	82° ± 2°		
SNB	80° ± 2°		
ANB	2° ± 2°		
Reconstructed Wits Index	0 ± 2 mm		
A-McNamara	Mixed dentition: 1 mm Permanent dentition: 0 mm		
Pog-McNamara	Years	Male	Female
	9	−6 mm	−8 mm
	12	−4 mm	−5 mm
	15	−2 mm	−2 mm
	>16	0 mm	0 mm
FH^Upper Inc	Hypodivergent		113° ± 1°
	Normodivergent		110° ± 1°
	Hyperdivergent		107° ± 1°
AnsPns^Upper Inc	Hypodivergent		113° ± 2°
	Normodivergent		110° ± 2°
	Hyperdivergent		107° ± 2°
SN^Upper Inc	103° ± 2°		
Upper Inc/A-Pog	3.5 mm ± 2 mm		
IMPA	Hypodivergent		93° ± 3°
	Normodivergent		90° ± 3°
	Hyperdivergent		87° ± 3°
Lower Inc/A-Pog	2 mm ± 2 mm		
Upper Inc^ Lower Inc	130° ± 5°		

Legend: SNA angle between Sella/Nasion/Maxillary Point A; SNB angle between Sella/Nasion/Mandibulary Point B; Pog: Pogonion; Inc: Incisor; FH: Frankfurt Plane; AnsPns: Palatal Plane; SN: Sella-Nasion Plane; IMPA: Mandibular Incisor/Mandibular Plane Angle.

**Table 2:** Articular Eminence Inclination (AEI) values in the right and left sides of the three groups.

	Male	Female	Tot.	Right AEI			Left AEI			*p*-Value ^a^
				Min.	Max.	Mean ± SD	Min.	Max.	Mean ± SD	
Class I	5	6	11	31.0°	59.5°	44.8° ± 9.0°	22.0	60.0	44.1° ± 10.8°	*p* = 0.527
Class II	6	7	13	28.2°	62.0°	42.0° ± 10.8°	29.5	64.0	47.0° ± 10.9°	*p* = 0.004 *
Class III	4	5	9	31.0°	51.5°	37.8° ± 6.7°	33.0	56.0	43.7° ± 7.8°	*p* = 0.020 *

Legend: Tot: Total; Min: Minimum; Max: Maximum; SD: Standard Deviation; ^a^ Friedman Test, * level of significance was set at *p* < 0.05.

**Table 3:** Kruskal–Wallis Test for right and left sides.

	Skeletal Class	Mean ± SD	Median	*p*-Value ^a^
Right AEI	Class I	44.8° ± 9.0°	45.5°	
	Class II	42.0° ± 10.8°	41.6°	
	Class III	37.8° ± 6.7°	38.4°	
				*p* = 0.174
Left AEI	Class I	44.1° ± 10.8°	47.0°	
	Class II	47.0° ± 10.9°	51.0°	
	Class III	43.7° ± 7.8°	44.0°	
				*p* = 0.624

Legend: SD: Standard Deviation; ^a^ Kruskal–Wallis Test; level of significance was set at *p* < 0.05.

## Data Availability

The data presented in this study are available on request from the corresponding author.

## Supplementary Materials

The following are available online at https://www.mdpi.com/article/10.3390/ijerph18115992/s1, Figure S1: Radiographical steps to obtain AEI measurement.
