# Supplementary material for: Evaluation of Articular Eminence Inclination in Normo-Divergent Subjects with Different Skeletal Classes through CBCT
Source: Int J Environ Res Public Health. 2021 Jun 3;18(11):5992. doi: 10.3390/ijerph18115992 (PMC8199764; doi:10.3390/ijerph18115992)
Supplement: Supplementary file 1 [file ijerph-18-05992-s001.zip › ijerph-1160688-supplementary.pdf]

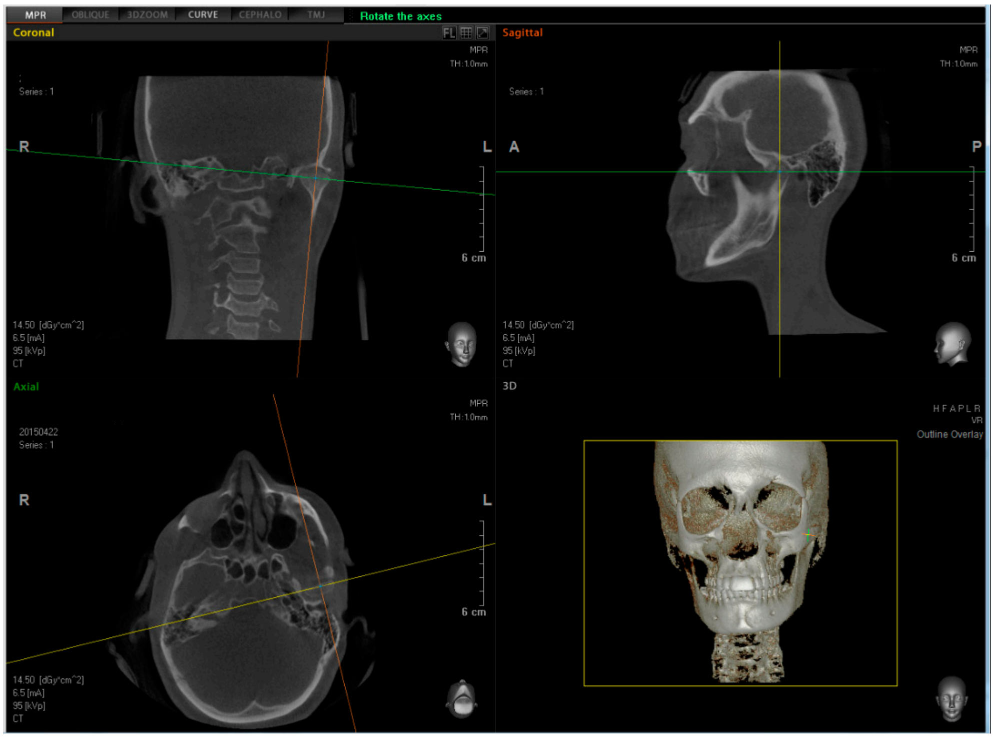

Figure S1. On the axial view, the section of the condylar process that had the widest mediolateral diameter was chosen as the reference view for reconstruction of the sagittal slices.

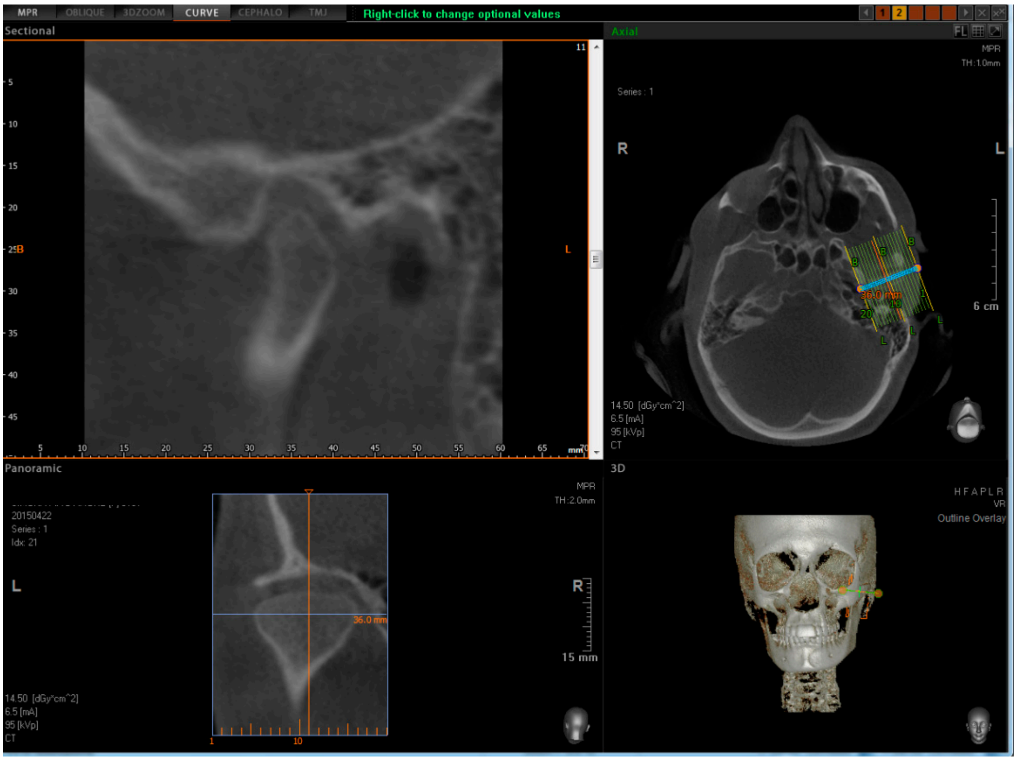

Figure S2. A line orthogonal to the mediolateral diameter of the condyle and parallel to the long axis of the condylar process was drawn.

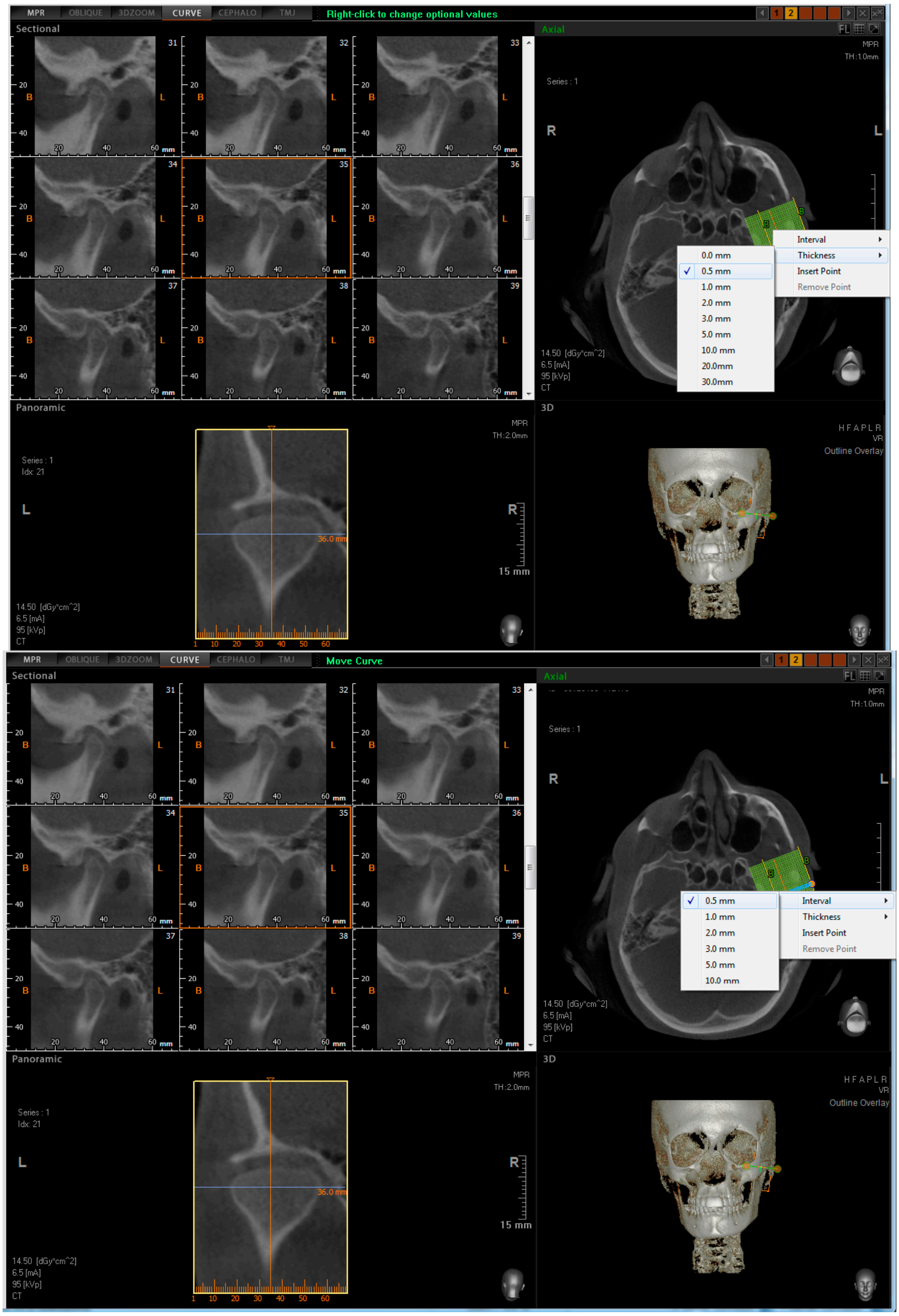

Figure S3. Sagittal images were reconstructed with a 0.5 mm slice interval/thickness.

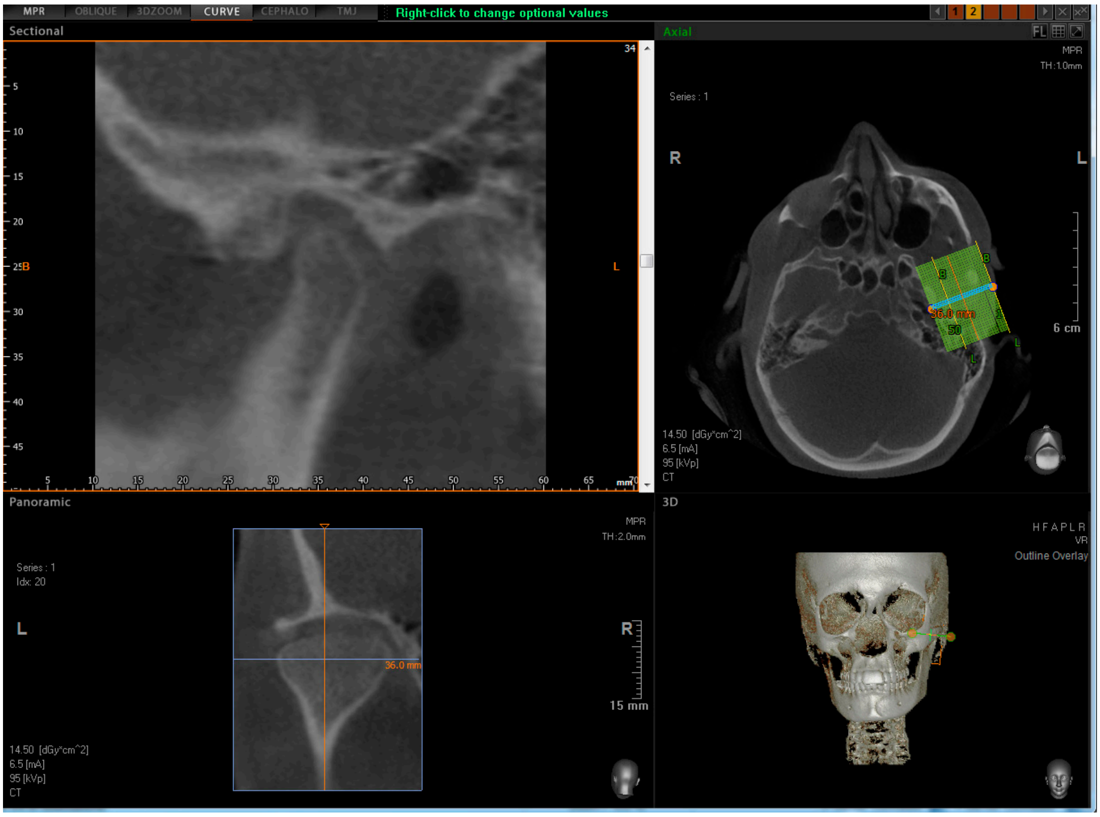

Figure S4. The measurements were established on the central sagittal section of the condyle.

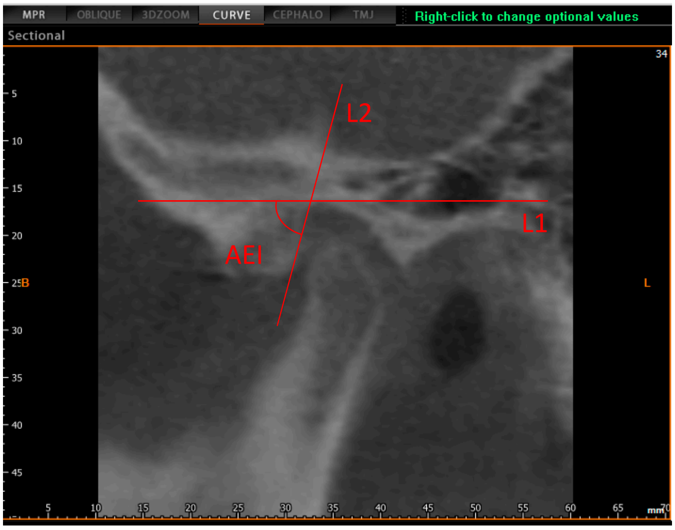

Figure S5. Two lines were traced:

- L1: a first horizontal line parallel to the Frankfurt Plane (FH) passing through the uppermost point of glenoid fossa;
- L2: a second line constructed along the posterior slope of the articular eminence, connecting the lowermost and most posterior point of the articular eminence and the uppermost and most anterior point of the glenoid fossa on the temporal bone.
